# Re: ChatGPT encounters multiple opportunities and challenges in neurosurgery

**DOI:** 10.1097/JS9.0000000000000749

**Published:** 2023-09-13

**Authors:** Qing-xin Yu, Rui-cheng Wu, De-chao Feng, Deng-xiong Li

**Affiliations:** a Department of pathology,Ningbo Clinical Pathology Diagnosis Center, Ningbo City, Zhejiang Province; b Department of Urology, Institute of Urology, West China Hospital, Sichuan University, Chengdu, Sichuan Province, People’s Republic of China

*Dear Editor*,

Large Language Models (LLMs) are attracting increased attention from researchers due to their capabilities and potential for adoption. Among these models, OpenAI’s ChatGPT stands out prominently. Kuang *et al*.^1^ delineated the merits and limitations of ChatGPT in the context of neurosurgery. We commend the authors for their innovative and impactful research. Upon meticulous scrutiny of their thought-provoking study, we endeavor to contribute our insights to enhance the scholarly dialogue surrounding this subject.

There exist several iterations of ChatGPT, each possessing distinct capabilities in data handling and question answering^2^. Among these iterations, ChatGPT3.5/4 is widely recognized. However, Kuang and colleagues omitted this essential information, potentially introducing bias into their study. Their research highlights ChatGPT’s inability to interpret and diagnose medical images. This limitation applies to ChatGPT3.5 but not to ChatGPT4, which demonstrates proficiency in summarizing radiology reports and significantly enhances Flesch reading ease score and Flesch–Kincaid reading level^3^. Furthermore, the authors assert that ChatGPT lacks the ability to address questions while taking into account a patient’s emotional state. We concur with this viewpoint, substantiated by our own study’s findings and practical experience. As a representative example of LLMs, ChatGPT prioritizes information accuracy and efficiency as its primary objectives. While the incorporation of humanistic considerations is of undeniable significance, it is an area that warrants enhancement in future iterations. Importantly, patients can readily access pertinent and accurate information through ChatGPT, facilitating informed decision-making. Additionally, patients have the opportunity to easily acquire surgical knowledge and pose questions prior to engaging with medical professionals, potentially enhancing effective doctor–patient communication^4^. In light of these considerations, the incorporation of the aforementioned suggestions may potentially enhance the methodological robustness of the study conducted by Kuang and colleagues^1^.

ChatGPT embodies a wealth of knowledge akin to an informed individual, necessitating its consideration as a collaborator in our clinical endeavors and research rather than merely a guideline searcher. Numerous studies have highlighted instances of ChatGPT generating inaccurate responses^5,6^. Employing a ‘think step-by-step’ approach has demonstrated a notable reduction in errors during mathematical reasoning tasks^7^. Analogous to the cognitive process of doctors, a sequential progression of logical inquiries is conducive to eliciting answers of enhanced quality. Engaging in discourse with ChatGPT, rather than solely soliciting specific surgical techniques, may offer a more comprehensive approach. Additionally, during these discussions, ChatGPT might offer innovative treatment suggestions by referencing the latest research findings. Consequently, it is prudent to view ChatGPT as a cooperative partner, particularly within surgical domains.

In aggregate, the endeavors of Kuang and colleagues yield novel and clinically significant findings. Our insights hold the potential to catalyze progress in this domain, augmenting the course of this discourse and presenting an alternate viewpoint for future explorations.

## Ethics approval

Not applicable.

## Consent

Not applicable.

## Sources of funding

No funding.

## Author contribution

Q.-x.Y., D.-c.F., R.-c.W., and D.-x.L.: collected and reviewed the literature and designed the writing; D.-x.L.: revised the manuscript. All authors read and approved the final manuscript.

## Conflicts of interest disclosure

All the authors declare that there are no relevant conflicts of interest to disclose.

## Research registration unique identifying number (UIN)

Not applicable.

## Guarantor

Qing-xin Yu and Deng-xiong Li.

## Data availability statement

Not applicable.

## Provenance and peer review

Not commissioned.
